# RNANet: an automatically built dual-source dataset integrating homologous sequences and RNA structures

**DOI:** 10.1093/bioinformatics/btaa944

**Published:** 2020-12-07

**Authors:** Louis Becquey, Eric Angel, Fariza Tahi

**Affiliations:** Université Paris-Saclay, Univ Evry, IBISC, Evry-Courcouronnes 91020, France; Université Paris-Saclay, Univ Evry, IBISC, Evry-Courcouronnes 91020, France; Université Paris-Saclay, Univ Evry, IBISC, Evry-Courcouronnes 91020, France

## Abstract

**Motivation:**

Applied research in machine learning progresses faster when a clean dataset is available and ready to use. Several datasets have been proposed and released over the years for specific tasks such as image classification, speech-recognition and more recently for protein structure prediction. However, for the fundamental problem of RNA structure prediction, information is spread between several databases depending on the level we are interested in: sequence, secondary structure, 3D structure or interactions with other macromolecules. In order to speed-up advances in machine-learning based approaches for RNA secondary and/or 3D structure prediction, a dataset integrating all this information is required, to avoid spending time on data gathering and cleaning.

**Results:**

Here, we propose the first attempt of a standardized and automatically generated dataset dedicated to RNA combining together: RNA sequences, homology information (under the form of position-specific scoring matrices) and information derived by annotation of available 3D structures (including secondary structure, canonical and non-canonical interactions and backbone torsion angles). The data are retrieved from public databases PDB, Rfam and SILVA. The paper describes the procedure to build such dataset and the RNA structure descriptors we provide. Some statistical descriptions of the resulting dataset are also provided.

**Availability and implementation:**

The dataset is updated every month and available online (in flat-text file format) on the EvryRNA software platform (https://evryrna.ibisc.univ-evry.fr/evryrna/rnanet). An efficient parallel pipeline to build the dataset is also provided for easy reproduction or modification.

**Supplementary information:**

Supplementary data are available at *Bioinformatics* online.

## 1 Introduction

A major part of any data-science work consists in finding appropriate data that contains enough signal to tackle the problem we are interested in. Then, cleaning the data to ensure uniformity of the measures, compatibility of the various data sources and protocols, and a reasonable amount of noise is sometimes the most time-consuming step.

Data-based methods have been popular for a long time in the field of RNA structural bioinformatics, for structure prediction (e.g. with Do *et al.*, 2006) or for mining and classification of interactions in motifs and networks (Petrov *et al.*, 2013; Reinharz *et al.*, 2018). Machine learning prediction methods start to rise [recent example with Singh *et al.* (2019)] following, with a few years delay, their rise in the field of protein structure prediction. However, past works use specialized datasets built on-purpose, probably using time and effort or databases focused on secondary structure only, for example. As we expect future developments in the field, in particular regarding 3D structure prediction, an annotated dataset of RNA 3D structures is needed. To our knowledge, the only available dataset is BGSU’s RNA Structure Atlas (https://rna.bgsu.edu/rna3dhub/pdb). It provides annotations of all RNA 3D structures in a browseable website, but their annotations cannot be downloaded as a whole and ready-to-use dataset for machine learning applications. Indeed, such dataset is needed to allow quick testing of new ideas, and to perform benchmarks and comparisons between future knowledge-based structure prediction algorithms.

We propose in this paper a standardized RNA dataset integrating and connecting together various pieces of information related to the RNA families, their sequences, their secondary and 3D structures. It is automatically built, allowing regular updates and improvements. This work was inspired by a recent publication by AlQuraishi (2019b), who proposed a standard dataset for protein structure prediction, called ProteinNet (mimicking the names of the famous WordNet and ImageNet reference datasets of vocabulary and images commonly used in machine-learning community). Our RNA dataset, accordingly called RNANet, differs from ProteinNet in several points. A much lower amount of available 3D data with a high proportion of identical chains (like ribosomal and transfer RNAs), as well as missing software tools adapted to RNA force us to forsake some pertinent ProteinNet strategies. Here, we propose a first attempt to build a usable dataset dedicated to RNA following the ProteinNet philosophy, using the currently available RNA data and tools. With the growth of databases and certainly successful use-cases in the future, the dataset will evolve, to reach, we hope, the size and quality of ProteinNet.

Specificities of RNA also make the corresponding dataset easier to build on some points. Among the key differences between RNA and proteins, we can cite the lower diversity of the residues: there are only four main nucleotides. When consensus residues exist, they are often related to structural features of the bases (purine or pyrimidine, weak or strong) rather than the physico-chemical properties that are used for amino-acids (acido-basicity, hydrophobicity). Then, it is easier to encode the three-dimensional conformation of a nucleotide, because the 3D descriptors are defined in the same way for all residues. This is not the case with amino-acids, one example being the number of torsion angles to consider differs depending on the considered amino-acid. The interactions between residues also are easier to encode. Most models consider that each base can interact one time on each of its 3 sides. This makes the interaction space of a nucleotide finite, while we have to consider many faces and rotamers when working with amino acids.

A common first problem in RNA computational biology is the prediction of structure from sequence. The basis for a dataset is then to gather sequences and their experimentally verified secondary structure and tertiary (3D) structure descriptors. A good start is to get the Watson–Crick basepairs, which permits to describe stems and various loops (hairpin loops, or multiple junctions).

**Fig. 1.**
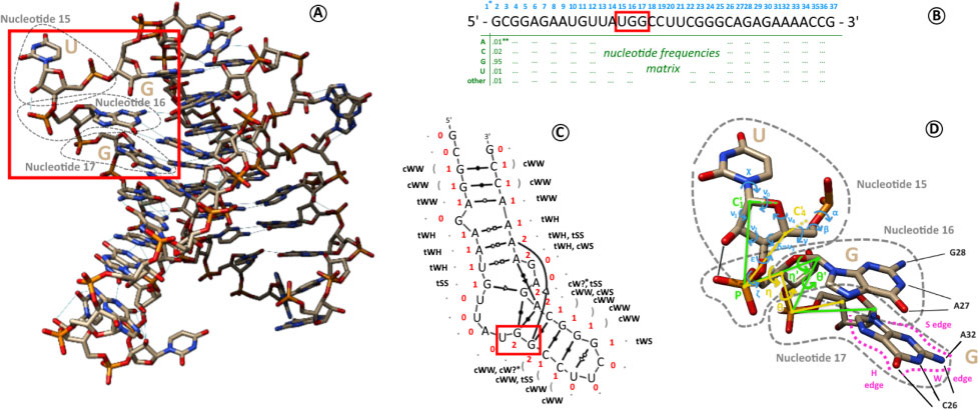
Different ways to encode the structural information of an RNA molecule. (**A**) Full-atom structure of an example RNA molecule (PDB 6sy6, chain D). (**B**) Primary sequence of this chain, using conventional A, C, G and U one-letter codes to describe commonly observed ensemble of atoms (the nucleotides). Residue numbers are indicated in light blue. When homologous sequences are available, it is possible to compute nucleotide frequencies at every position (a.k.a. position-specific scoring matrices or PSSMs). (*) Residue n^∘^1 is not resolved in 3D and not represented on the figure. (**) The example values are fictive since 6sy6-D does not belong to a particular RNA family to our knowledge. (**C**) Secondary structure graph of the chain, with the number of base contacts for each base (in red), the base symbol in dot-bracket notation (light gray) and the non-canonical interactions between bases in Leontis–Westhof nomenclature (in black). The first letter *c* or *t* indicates a *cis* or *trans* configuration, and the two following uppercase letters H, S and W, are the base’s sides interacting (illustrated on nucleotide 17 in D). (*) A question mark (?) is used when the nucleotide interacts with only one atom, which is not enough to define a base side. (**D**) Detailed 3D geometric descriptors illustrated on nucleotides 15, 16 and 17 of the chain. Nucleotide 15 is annotated with its torsion angles (in light blue). Nucleotide 16 is annotated with virtual bond systems and their pseudotorsion angles *η* and *θ* (in yellow) and η′ and θ′ (in green). Nucleotide 17 is annotated with a label on each base edge: H on the Hoogsteen edge, W on the Watson–Crick edge, S on the sugar edge. These three residues are highlighted by a red box in (A), (B) and (C)

Then, to capture the 3D structure, two kinds of descriptors can be used in complementarity: those which focus on the backbone configuration (helix forms, ribose configurations, torsion angles), and those which focus on all the base–base and base-backbone interactions [the 12 variants of Leontis–Westhof base–base pairing nomenclature (Leontis and Westhof, 2001), plus stacking interactions and base-phosphate interactions]. The different levels of approximation of an RNA structure are illustrated onFigure 1. The full-atom 3D structure in Figure 1A can be encoded, as a first approximation, into a sequence of letters, the primary structure (Fig. 1B), because we know, by convention, to which composition of atoms each of the letters corresponds. The next step is to describe the interactions between each of the bases. Such description is proposed on Figure 1C, with the secondary structure graph of the same molecule and its non-canonical interactions. Yet, the global shape and folding of the polymer appear. The finest level of description is to describe every atom’s position using geometrical parameters. On Figure 1D, we annotate the three nucleotides highlighted by a red rectangle on Figure 1A, B and C using geometrical descriptors.

Finally, on top of the structural descriptors of each RNA chain, the most recent developments in structural bioinformatics have shown the importance of capturing the folding information of macromolecules families, so that the family fold can be used later when trying to predict how one sequence will fold in space [e.g. Xu, (2019) and AlQuraishi (2019a) with proteins, or Magnus *et al.* (2019) in the field of RNA]. Therefore, it is useful to include position-specific scoring matrices computed on homologous sequences in addition to every chain sequence, to reflect diversity or conservation inside a family of molecules. For that reason, one of the key elements of the dataset, which brings novelty to this work, is to provide nucleotide frequencies at every position of the RNA 3D structures from the PDB (as illustrated below the sequence on Fig. 1B).

To summarize, for each RNA chain, we provide descriptors about the sequence and sequence variation, about the secondary structure and pairing scheme of each nucleotide (with non-canonical interactions and multi-basepairs), and fine descriptors of the 3D conformation (angles, torsions). We provide the data in an SQLite database and flat text format (CSV files) to be opened in a spreadsheet or parsed by user scripts. The dataset is updated every month to take into account newer data.

In the next section, we go through the steps of the pipeline, starting from the public databases, to the final dataset. We explain the choices made and why it differs from ProteinNet. We then provide some descriptive statistics about the obtained data and its quality in Section 3.

## 2 Materials and methods

**Fig. 2.**
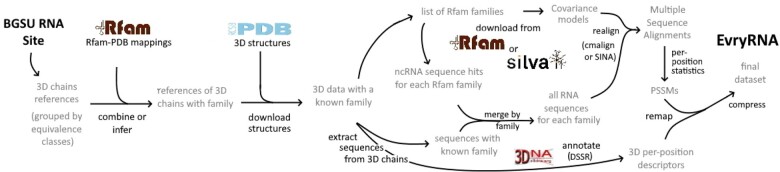
Pipeline of the RNANet dataset construction from public databases PDB, Rfam and SILVA

To build the RNANet dataset, we developed a pipeline whose different stages are summarized in Figure 2. The pipeline gathers a list of RNA chains from the BGSU RNA research group website (Leontis and Zirbel, 2012), and searches for mappings of these chains to Rfam RNA families (Kalvari *et al.*, 2018). The identified mappings allow then to download known homologous sequences and realign them with the 3D chain sequences, to get sequence variation statistics. In parallel, three-dimensional structures are downloaded and annotated by DSSR (Lu *et al.*, 2015) to yield descriptors of the 3D structure. Finally, the two kinds of per-position information are re-mapped one to each other, and the dataset is saved. We thus obtain a list of various nucleotide descriptors at each scale: sequence and its variability, secondary structure and non-canonical interactions and precise geometrical descriptors, including sugar puckering and torsion angles. The complete list is presented in Supplementary Table S1 in Supplementary Material. Details on the choices made for each of the steps are given in the following sections.

### 2.1 Data selection

The first step is to identify RNA chains of sufficient quality in the 3D data available in the PDB. To do so, we use the list kept up-to-date by the BGSU RNA research group (Leontis and Zirbel, 2012). We include all the RNA chains with resolution better than a desired resolution threshold (4.0 Angströms by default, which can be changed using a command-line option). The number of available RNA chains as a function of structure resolution is presented in Supplementary Figure S3. Note that we keep the redundant chains. This is motivated by the lack of data: as we have few 3D data, we need to exploit every variant of the same molecule if several structures are available. Because replica of crystallography or EM experiments may vary a little in the position of nucleotides, interactions and 3D descriptors like angles or sugar pucker may also vary a little. This gives information about the probability distributions of these parameters. Redundancy could be eliminated or controlled later by the user, by further re-filtering the final dataset. The BGSU list is not exhaustive of the RNA chains available in 3D (it lacks, for example, all the available NMR structures). But, the RNA chains in the BGSU list are organized by redundancy in so called ‘equivalence classes’, and we find it useful to identify more links between 3D chains and homologous sequences.

An important bottleneck is to identify homologous sequences for all these chains, so that we can compute nucleotide frequencies at every position in the chain. A good strategy proposed in AlQuraishi (2019b) for proteins is to use the jackHMMER tool (Johnson *et al.*, 2010), which performs an iterative search of homologous sequences against wide sequence databases. Unfortunately, the tool does not support nucleic acid sequences. However, the HMMER software tools, excluding jackHMMER, have been specialized for DNA/RNA sequences resulting in the Infernal tools (Nawrocki and Eddy, 2013). These tools work with the pre-computed covariance models of the Rfam RNA families (Kalvari *et al.*, 2018). With every Rfam family comes a covariance model and a list of sequence hits from reference genomes. Infernal can realign sequences with the covariance model to get a multiple sequence alignment. Moreover, Rfam provides a list of 5567 mappings between PDB structures and Rfam families, which is why we decided to use it as our basis to get homologous sequences. Using them may not be the way leading to the highest number of hits between the existing 3D chains and homologous sequences. Indeed, Rfam is evolving since release 13.0 to a high-quality database based on non-redundant genomes and probably lacks some species and ‘families’. We may detect more homologous sequence clusters by scanning more genomes ourselves. However, we do not wish to compete with Rfam, and prefer to rely on their expertise and regular updates. Hence, we filter the previous list of 3D RNA chains to keep only those with an available mapping to an Rfam family, namely, a covariance model. We also retain 3D structures for which no mapping is directly available, but a mapping exists to another 3D chain from the same equivalence class in the BGSU redundancy list. Considering all the data without a resolution requirement, the filtering reduces the number of chains from 11 702 to 6282 (53%). When no mapping from Rfam is available, but several mappings to different families can be inferred using the equivalence class, the chain is copied and truncated to save the portion corresponding to each mapping, respectively. This procedure increases the counter to 6668 chains, including the copies of chains that are mapped several times. Optionally, as the Rfam-PDB mappings are relative to portions of PDB chains, the user can provide an option in the command line to extract the desired portions only from the source mmCIF files, excluding the other uninteresting chains, waters, ligands and ions.

### 2.2 Annotation of the 3D chains

Now that we have a collection of data points (one datum corresponding to one RNA chain), we need to use an annotation program to provide a list of descriptors of these chains. The list of RNA-containing mmCIF files is therefore fed to the RNA annotation software tool DSSR (Lu *et al.*, 2015), to extract position-specific information at every nucleotide. This includes nucleotide sequence, with DSSR’s wide support of modified bases, base–base interactions in both DSSR and Leontis–Westhof (Leontis and Westhof, 2001) nomenclatures and with support of multiple interactions per base. This also includes geometric descriptors of the bases, riboses and backbone: first, the six torsion angles of the backbone, commonly denoted by *α*, *β*, *γ*, *δ*, *ϵ* and *ζ*; then, the ribose-base torsion angle *χ*, and the associated label ‘syn’ or ‘anti’; and then, the five torsion angles of the ribose, commonly denoted by *ν*_0_ to *ν*_4_. Then come the pseudo torsion angles of virtual bond systems *η*/*θ*, *η*′/*θ*′, *η*″/*θ*″, summarizing the backbone with only P and C4′ atoms for *η*/*θ*, P and C1′ for *η*′/*θ*′ or P and base center position for *η*″/*θ*″ [see Keating *et al.*, (2011) for a good introduction to the backbone description systems]. DSSR also provides the phase angle of the ribose cycle and its amplitude, and the associated ‘sugar puckering’ label. Two descriptors concern the position of the 3′-phosphorus. Finally, if the nucleotide is involved in a stem, the stem type (A, B or Z) is reported. A detailed description of computed 3D descriptors is available in the third group of Supplementary Table S1 in Supplementary Material. Note that using the whole mmCIF structures for annotation permits to yield both intra-chain and inter-chain interactions in the descriptors. The ‘pair_type’ fields contain information about both. But the ‘paired’ field, which gives the index value of the corresponding nucleotide for intra-chain interactions, is set to zero if an interaction is inter-chain.

### 2.3 Alignment of the sequences

As the RNA sequences of the chains have now been extracted using DSSR, we can now compute position-specific nucleotide frequencies. To do so, we use the previously retrieved PDB-Rfam mapping of every chain. We gather together all the sequence hits of a given Rfam family, plus the sequences of the 3D chains that are mapped to this family, in a single file. A multiple sequence alignment is then recomputed using Infernal’s *cmalign* program. Technical limits (in particular the *cmalign* memory requirements) make it impossible to realign the largest families on regular computer hardware. It is the case of the ribosomal sub-units [families from Rfam clans CL00111-SSU (Small Sub-Units) and CL00112-LSU (Large Sub-Units)]. So, we decided to re-align these sequences differently. Instead of using Infernal and Rfam sequences, we consider the specialized database SILVA (Pruesse *et al.*, 2007) and its aligner *SINA* (Pruesse *et al.*, 2012). The SILVA data are larger than Rfam hits on the above families’ covariance models, hand-curated, and their specialized aligner is faster and uses less memory. As ribosomal RNAs are a consequent portion of the whole RNA 3D dataset, it is worth using specialized tools. Both *SINA* and *cmalign* output multiple-sequence alignments. Multiple sequence alignments are then summarized into position-specific scoring matrices (PSSM), which basically are a summary of the base counts per column in the alignments.

### 2.4 Re-mapping strategy

The final step is to merge the per-position data concerning the RNA 3D chain with the per-position nucleotide frequencies in the multiple sequence alignments. As each sequence extracted from a 3D chain has been realigned with its homologs, it now contains gaps in its aligned version. On the 3D chain side, gaps may only occur if some residues are not resolved. On the multiple sequence alignment side, *cmalign* produces two kinds of gaps: gaps to the family consensus whose symbols are ‘-’ and describe an important deletion with respect to the family pattern, and gaps whose symbols are ‘.’ that are just padding because another sequence contains an insertion with respect to the family consensus. Starting with the two different gapped sequences, our mapping strategy is the following:

- If the two sequence symbols (nucleotide letter or gap) match, we merge the position descriptors, and move to the next position.
- If the 3D chain contains a gap (‘-’ symbol) but does not face a gap to the consensus in the multiple alignment, we first try to search for a real gap to the consensus in the following positions (ignoring insertion gaps ‘.’ until a resolved nucleotide letter comes). If one is found, we map the two positions together. If none is found, we search for an insertion gap ‘.’. If no gap is found at all, we map the 3D chain descriptors with unknown homology descriptors (NaN values). This happens when the alignment is locally incorrect.
- If the aligned sequence contains a gap, but not the 3D chain, we just skip this position and move to the following.
- If the sequence symbols do not match but are not gaps, we throw an error. This never happens in practice.

### 2.5 Special attention paid to specific cases

In order to maximize the amount of data, problematic cases were not discarded. We try to overcome particular situations as much as possible to include all the data. First, it is very common to observe missing residues in RNA chains, because of unresolved portions in the mmCIF file. Their positions are marked as gaps in the sequence. By default, they are later replaced by the most common nucleotide at this position among the homologous sequences in a dedicated database field (nt_align_code). A gap character ‘-’ remains in the main field (nt_code, see Supplementary Table S1). This can be turned off with an option. Another common case is the presence of ligands and small molecules crystallized with the RNA chain. DSSR often detects them as residues and tries to annotate them like nucleotides. This leads to wrong ‘modified bases’ (which are not bases at all) at the end of chains. We automated the detection and removal of such ligands, but unfortunately we cannot guarantee that all were correctly detected. We also tackle various particular cases where nucleotides are numbered in a non-standard way in the mmCIF files, e.g. using letters, negative numbers, numbers augmented by a thousand to indicate an artificial nucleotide in the biological sequence, and many others. Such anomalies are very common. Our processed chains are numbered from one to their length. Finally, we do not remove base–base interactions which do not fit in one of the Leontis–Westhof nomenclature descriptions. This concerns, for example, bases interacting using only the Guanine *N*_2_. We include them, but label them in a group named ‘other’.

### 2.6 Implementation details

RNANet is built automatically using a Python script available on the EvryRNA website (https://EvryRNA.ibisc.univ-evry.fr/evryrna/rnanet). It heavily relies on the BioPython package (Cock *et al.*, 2009) to parse mmCIF and alignment files. It depends on a working installation of *SINA*, *DSSR* and *Infernal*. A Docker container is provided to ease the installation. Running it on a multi-core computer greatly speeds up the computation, but requires more memory, at scale. Fifteen hours are required to compute all the dataset for the first time on a 32-core server (Intel Xeon E7-4850 v4 @32x2.10 GHz), with 48 GB of RAM. Later runs will, by default, only perform necessary computations to keep the database up-to-date more quickly.

## 3 Results and discussion

### 3.1 Dataset files and database

The pipeline organizes the information in a collection of tables in an SQLite3 database (the database scheme is provided in Supplementary Fig. S1). This makes it easy to query specific data. By default, all the per-position information is extracted to CSV files, one per RNA chain. It is possible to re-extract a different set of files using for example a different structure resolution threshold. We provide a few example using Python and the sqlite3 package on EvryRNA.

Another example of filtering criterion is the data publication date. With ProteinNet, AlQuraishi proposes pre-computed versions of his dataset with only the available data at some points in time, corresponding to rounds of the CASP protein-folding competition. This allows users to compare their prediction methods or tools with all the popular ones that have been participating a past CASP competition. Users might want to do the same with the RNA-Puzzles problems (Miao and Westhof, 2017). No time-frozen releases of the dataset are pre-computed, but it is possible to query our SQLite database to yield only the chains whose release date is earlier than a certain RNA-Puzzles problem.

### 3.2 General statistics over the dataset

Using the SQL database, it is easy to query various statistics over the available RNA structural data to date. For example we can query the most common RNA family, which is RF00005 corresponding to transfer-RNAs, with 1694 chains.

To date (September 2020) and without further filtering based on structure resolution, we registered 10 173 RNA 3D chains available in the PDB. Rfam provided 5505 mappings to known families (54%). Using BGSU’s redundancy lists (v3.145), we extended this list to 6217 unique RNA chains, or 6602 chains if we accept to map copies of chain portions to different Rfam families. These figures are slightly lower than the ones given in Section 2.1, because they exclude chains that are too short, contain no nucleotides (backbone only), or are incorrectly parsed by DSSR. The chains were mapped to 92 Rfam families. The number of homologous sequences found by family ranges from 5 (RF00044) to 1 429 931 (tRNAs from Rfam, RF00005), or 2 225 272 (SSU sequences from SILVA). Detailed numbers of families as a function of the resolution threshold considered, and numbers of sequences mapped to each Rfam family are given in Supplementary Figure S2 and Supplementary Table S2 in Supplementary Material.

**Fig. 3.**
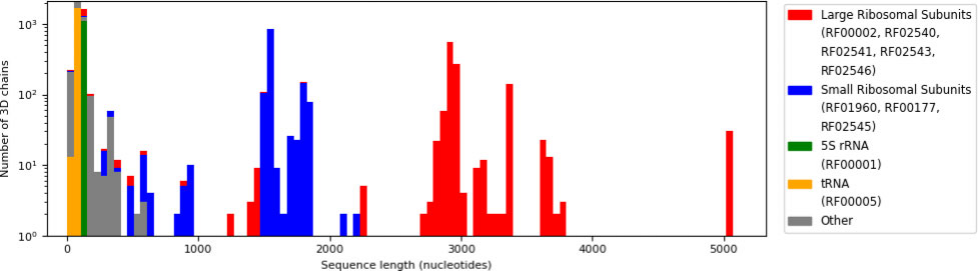
Distribution of sequence lengths among RNA families (logarithmic scale). The longest families are the 4 LSU families (RF02540, RF02541, RF02543 and RF02546 for archaeal, prokaryotic, eukaryotic and mitochondrial ribosomes, respectively) with over 2500 nucleotides. SSUs follow (RF00177, RF01960 and RF02545), around 1600 nucleotides long. Transfer RNAs are the most common family, but they are less than 300 bases. The group labelled ‘Other’ contains various RNAs from 82 families, all smaller than 500 nucleotides long

Concerning the nucleotide distribution over the RNA families, Figure 3 presents both a histogram of RNA chain lengths, and the distribution of Rfam families within that histogram. As we can see, the longest chains are from Large ribosomal Sub-Units (LSU), followed by the Small Sub-Units (SSU). In general, the dataset is mainly composed of ribosomal RNAs, which also are the longest chains, followed by transfer RNAs. The majority of other RNA families are represented by fewer chains, and are often less than 500 bases long. Nucleotide frequency statistics by RNA family are provided as an additional CSV file to download on EvryRNA.

**Fig. 4.**
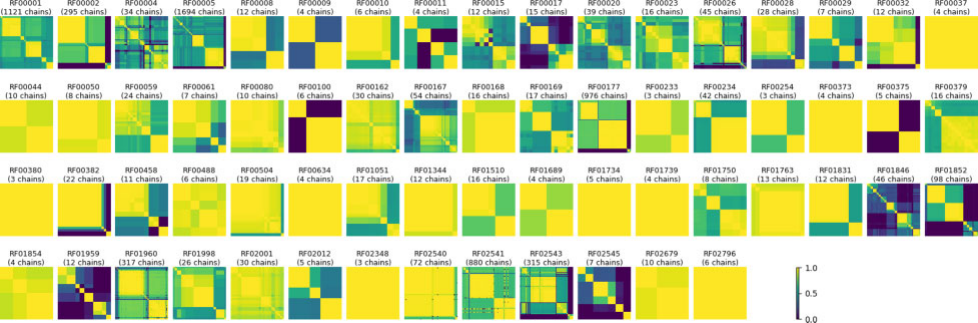
Sequence identity matrices of RNA chains mapped to various Rfam families (clustered by Ward’s method). The more yellow (or light gray) a distance matrix is, the more similar the sequences are. The more blue (or dark gray) the matrix is, the more distant the sequences are. Families with less than three chains available in 3D are not represented (20 families)

To quantify the redundancy among RNA families, we compute sequence identity matrices for every Rfam family. The sequence identity matrices can also be used to perform a hierarchical clustering over chains. The result is presented graphically on Figure 4. As expected, a majority of the 92 families consists of a very few, or very similar sequences. A high average identity in a RNA family is reassuring regarding the quality of the sequence alignment, but also is a sign of great redundancy in the sequence space at least. However, the RNA chains which share a common sequence may present different secondary and 3D structures, since the sequence does not completely determine the structure. We do not further filter the data to eliminate redundancy (yet), but users are encouraged to do so by themselves, at the level of redundancy they chose to keep, and at the cost of data reduction.

### 3.3 Joint distribution of the *η*/*θ* pseudotorsions

**Fig. 5.**
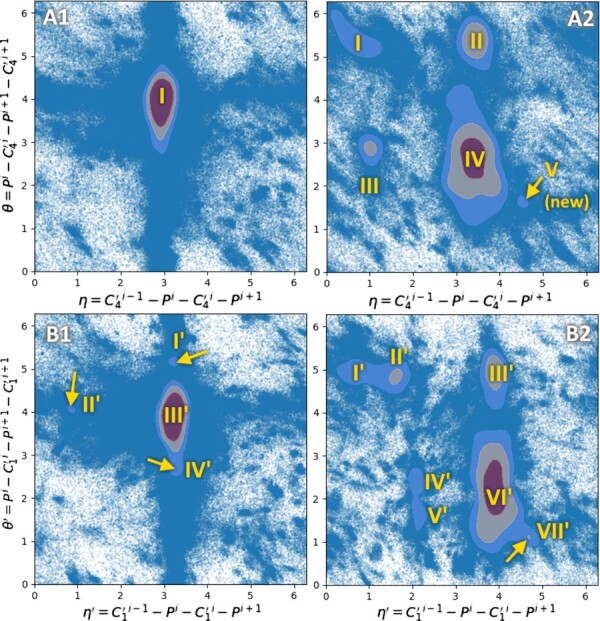
Ramachandran-like plots of the couples of pseudotorsions (η,θ) in A1 (non-helical C3′-endo nucleotides) and A2 (C2′-endo nucleotides), and (η′,θ′) in B1 (non-helical C3′-endo nucleotides) and B2 (C2′-endo nucleotides). Gaussian kernel density estimates are superposed to scatter plots. The line contours correspond to ρ+σ, ρ+2σ and ρ+4σ where *ρ* is the average height of the kernel and *σ* its standard deviation. The used nucleotides are from unique chains of resolution 4.0 Å or better. Plots of the distributions using more restrictive thresholds at 3.0 and 2.0 Å are available in Supplementary Section S4

In Wadley *et al.* (2007), the authors identified that non-helical nucleotides usually have discrete possible conformations. To show evidence, they measured and plotted the joint distributions of the pseudotorsion angles *η* and *θ* in non-helical nucleotides, like Ramachandran did with amino-acids in proteins. For a nucleotide *i*, *η_i_* is defined as the torsional angle between the four atoms $C\prime_{4}^{i-1}$, *P^i^*, $C\prime_{4}^{i}$ and $P^{i+1}$. Similarly, *θ_i_* is defined as the torsion angle between *P^i^*, $C\prime_{4}^{i},\;P^{i+1}$ and $C\prime_{4}^{i+1}$. These pseudotorsions have been proven easier to use than the six regular torsion angles of the RNA backbone (Duarte and Pyle, 1998). They propose a list of clusters linked to a particular conformation of the nucleotides. As an example use case of the dataset, we try to reproduce the same clusters with the 2020 data. The joint distribution plot of the *η*/*θ* pseudotorsions in our dataset is given on Figures 5A1 and A2, to compare with Figure 6a and b of Wadley *et al.* (2007) respectively. With the growth of the amount of solved structures, all of the C3′-endo clusters except the large central one have vanished. However, we still find the C2′-endo clusters, plus a small new fifth one.

The simplest hypothesis about the difference of clusters between Figure 5A1 and the original plot from Wadley might be the growth of the amount of data dissolving small peaks that were significant in 2007 in a widespread mass of points. We indeed can observe the old clusters if we decide to relax the average plus one standard deviation threshold to only average, for example (data not shown). This threshold was originally proposed by Wadley *et al.* to focus on significant peaks. Nevertheless, the proportion of these small peaks relatively to the large central one has changed over the years. The second hypothesis could be a difference in the way we filter non-helical nucleotides, which fall in the central region. We indeed removed nucleotides annotated by DSSR as being part of an A-form, B-form or Z-form 3D stem. As many nucleotides may not fulfil DSSR’s detection criteria, but still be part of or near a helix, they are included and participate to the massive peak.

A few years later, Keating *et al.* proposed to use another definition of the pseudotorsions, called η′ and θ′, which uses carbons $C\prime_{1}$ instead of $C\prime_{4}$ (Keating and Pyle, 2010). Indeed, because base planes are easy to solve, $C\prime_{1}$ are the most precisely located atoms in X-ray crystallographic RNA structures. So we recompute the plot with η′ and θ′, and the results are given on Figure 5B1 and B2, for non-helical $C\prime_{3}$-endo and $C\prime_{2}$-endo nucleotides respectively. Here, we observe slightly different cluster positions, including small peaks on the C3′-endo plot, pointed by arrows on Figure 5B1. They are well conserved compared to the 2007 dataset.

### 3.4 Shortcomings compared to ProteinNet

Besides the fact that the inspiration for RNANet came from ProteinNet, the two are actually different, because of methodology variations and because of the gap in the available amount of data between proteins and RNA.

#### 3.4.1 Small amount of data

The convenience of the Rfam/Infernal ecosystem, providing precomputed hits of non-coding RNA sequences on the families covariance models, comes at the price of heavy data reduction. Because a mapping is not (yet) available for every 3D chain, more than a third of the available chains are unused. This can be critical to deep-learning models. The development of a nucleic-acid equivalent of jackHMMER (Johnson *et al.*, 2010), used in ProteinNet to find sequence homologs (AlQuraishi, 2019b), could be of great use. First, it would allow to take into account all the available 3D chains. Second, it would find more homologous sequences for every chain, using even larger RNA databases (Rfam is built only on a collection of reference genomes as a basis, and only non-coding RNAs).

#### 3.4.2 Data diversity and pre-computed splits of training, testing and validation sets

As Figure 4 shows, sequences are very close to each other inside a family. And even across several families, sequences are dependant on each other and linked by evolutive relations, e.g. the three families of large ribosomal sub-units in the three great living domains. So, without further filtering, RNA chains of the dataset cannot be considered independent and identically distributed. This also means that the dataset cannot be naively split randomly to get training and test sets. To overcome this, the ProteinNet approach (AlQuraishi, 2019b) proposes to carefully select sequences to build training and test subsets without having two sequences with too much similarity in one of each. This would indeed constitute a leak of information between the training and test sets. Such work is even harder with RNA. First, we cannot reproduce the same filtering algorithm yet, because the tools used for the multiple-sequence alignment based clustering, HHBlits (Remmert *et al.*, 2012) and MMSeqs2 (Steinegger and Söding, 2017), do not support nucleic acid sequences for now. As for jackHMMER, support of these tools for nucleic acid sequences would allow similar strategies. Second, any other automated or manual approach would still be difficult, because of the small amount of data. As a consequence, we have to provide the un-split dataset for now, and let the users decide how they want to build the splits. Splits may come in a later release if a pertinent strategy is found, or if new software tools are released.

### 3.5 Upcoming plans and new descriptors

RNANet will be continuously updated to integrate newer data as the PDB grows. The first thing to do, as soon as the data permits it, would be to further cluster all the RNA chains by similarity and define sets of points with enough distance between them to be considered independent. This would allow pre-computed training, testing and validation sets.

Moreover, if an equivalent of the protein tools jackHMMER, HHBlits or MMSeqs2 is released with support for RNA sequences, improvements would become possible to closer follow the ProteinNet strategy. A sequence-alignment-based clustering step could then be added, and the search for homologous sequences upgraded.

In addition, more descriptors would be easy to compute and could be added in future versions. For example, the BGSU RNA Structure Atlas which uses annotations by FR3D (Sarver *et al.*, 2007) instead of DSSR does not compute the 3D geometric descriptors, but proposes annotations of stacking interactions, base-phosphate interactions, and loop types (hairpin, internal or multiple loops). DSSR also proposes a way to identify splayed-apart conformations of some nucleotides, which could be integrated to our analysis. Such features might be added in a future release of the dataset.

## 4 Conclusion

To speed up the development of machine-learning based RNA structure prediction tools, we developed a pipeline which builds a multi-scale dataset, called RNANet, from public data. Both the dataset and pipeline code are available on the EvryRNA software platform (https://EvryRNA.ibisc.univ-evry.fr/evryrna/rnanet).

The dataset gathers information about the sequences, the secondary structures and the tertiary structures of available RNA chains chosen with resolution better than a given threshold. We propose a list of various descriptors, including nucleotide frequencies computed on sequences from the Rfam and SILVA databases known to be homologous to the chains, realigned with the 3D chains sequences.

The dataset can be easily sub-sampled or filtered on demand using custom SQL queries. For example, structures available before a certain date can be retrieved, to allow comparisons with previous RNA-Puzzles benchmarks (Miao and Westhof, 2017). A structure resolution criteria, or a gap percentage threshold, or a minimum number of certain types of basepairs can also be applied to yield only the required data.

The amount of data, being directly dependant on the content of public databases like the PDB, is for now an obvious limit when it comes to deep models, which would be subject to over-fitting issues. In the meantime, the dataset is useful to compute descriptive statistics about RNA structures [e.g. reproducing (Wadley *et al.*, 2007) results, motif discovery or recurrent interaction patterns description]. Simple supervised learning models, trying to predict some of the structural descriptors using sequence and homology data, can still make a great use of RNANet. The automated pipeline will keep the RNANet dataset up-to-date on EvryRNA, as long as the public databases grow.

## Supplementary Material

btaa944_Supplementary_Data
