# Supplementary material for: RNANet: an automatically built dual-source dataset integrating homologous sequences and RNA structures
Source: Bioinformatics. 2020 Dec 7;37(9):1218–24. doi: 10.1093/bioinformatics/btaa944 (PMC8189678; doi:10.1093/bioinformatics/btaa944)
Supplement: btaa944_Supplementary_Data [file btaa944_supplementary_data.pdf]

1 Description of the RNANet database

Table 1 lists all the per-nucleotide (per-position) descriptors available in the database. Figure 1 shows the associated SQL schema.

Descriptor	Label	Type
Index of the residue in the chain (from 1 to N)	index_chain	int ≥ 1
Index of the residue in the source mmCIF file	nt_resnum	int ≥ 1
Position of the nucleotide in the chain, normalized by its length (value between 0 and 1)	nt_position	float
Nucleotide name, including modified bases (like 5MC)	nt_name	str
One-letter name. Lowercase "acgtu" letters are used for modified "ACGTU" bases	nt_code	char
Letter used for sequence alignment (*)	nt_align_code	char
One-hot encoded sequence. 'other' contains gaps, unknown and modified nucleotides	is_A, is_C, is_G, is_U, is_other	0 or 1
Nucleotide frequencies (PSSM) at the current position in this RNA family	freq_A, freq_C, freq_G, freq_U, freq_other	float
Secondary structure in dot-bracket notation of this position	dbn	char
Zero, or comma-separated values of index_chain of the nucleotide(s) which is(are) paired with this one. Canonical (Watson-Crick or Wobble) basepairs are first in the list.	paired	int, int, ...
Number of other bases interacting with the nucleotide	nb_interact	int ≥ 0
Type of basepair in Leontis-Westhof nomenclature (comma-separated list)	pair_type_LW	str, str, ...
Type of basepair in DSSR nomenclature (comma-separated list)	pair_type_DSSR	str, str, ...
The six torsion angles of the backbone, from 5' to 3', between 0 and 2π	alpha, beta, gamma, delta, epsilon, zeta	float (rad)
Difference between epsilon and zeta torsion angles	epsilon_zeta	float (rad)
Conformation of the backbone	bb_type	BI, BII, or '..'
χ torsion angle (between ribose and base)	chi	float (rad)
Conformation of the sugar with respect to the base (depends on χ)	glyco_bond	syn or anti
Torsion angles of the ribose cycle	ν_0, ν_1, ν_2, ν_3, ν_4	float (rad)
If the nucleotide is involved in a stem, the stem type	form	A, B, Z or '.'
Z-coordinate of the 3' phosphorus atom with reference to the 5' base plane	ssZp	float
Perpendicular distance of the 3' P atom to the glycosidic bond	Dp	float
Pseudotorsions between P and C'_1	eta, theta	float (rad)
Pseudotorsions between P and C'_4	eta_prime, theta_prime	float (rad)
Pseudotorsions between P and the base center	eta_base, theta_base	float (rad)
Conformation of the ribose cycle	phase_angle	float (rad)
Amplitude of the sugar puckering	amplitude	float
Conformation of the ribose cycle (10 classes corresponding to specific ranges of phase)	puckering	str

Table 1: Descriptors of the data points (RNA chains), concerning their sequence (top group of the table), secondary structure (second group) and 3D structure (bottom group). The bottom group directly comes from annotation of the available 3D structure by DSSR. Base-pairs and their types are also the ones detected by DSSR, but the numbering of the base partners has been modified to fit our own `index_chain` numbering instead of the structure residue numbers. The homology data (frequencies) are computed on the multiple sequence alignments. When a value is missing, it is set to 'NaN'. (*) The nt_align_code field is set twice: first when annotating the 3D structure with DSSR, and then after the re-mapping step when gaps are replaced by the most frequent base at this position.

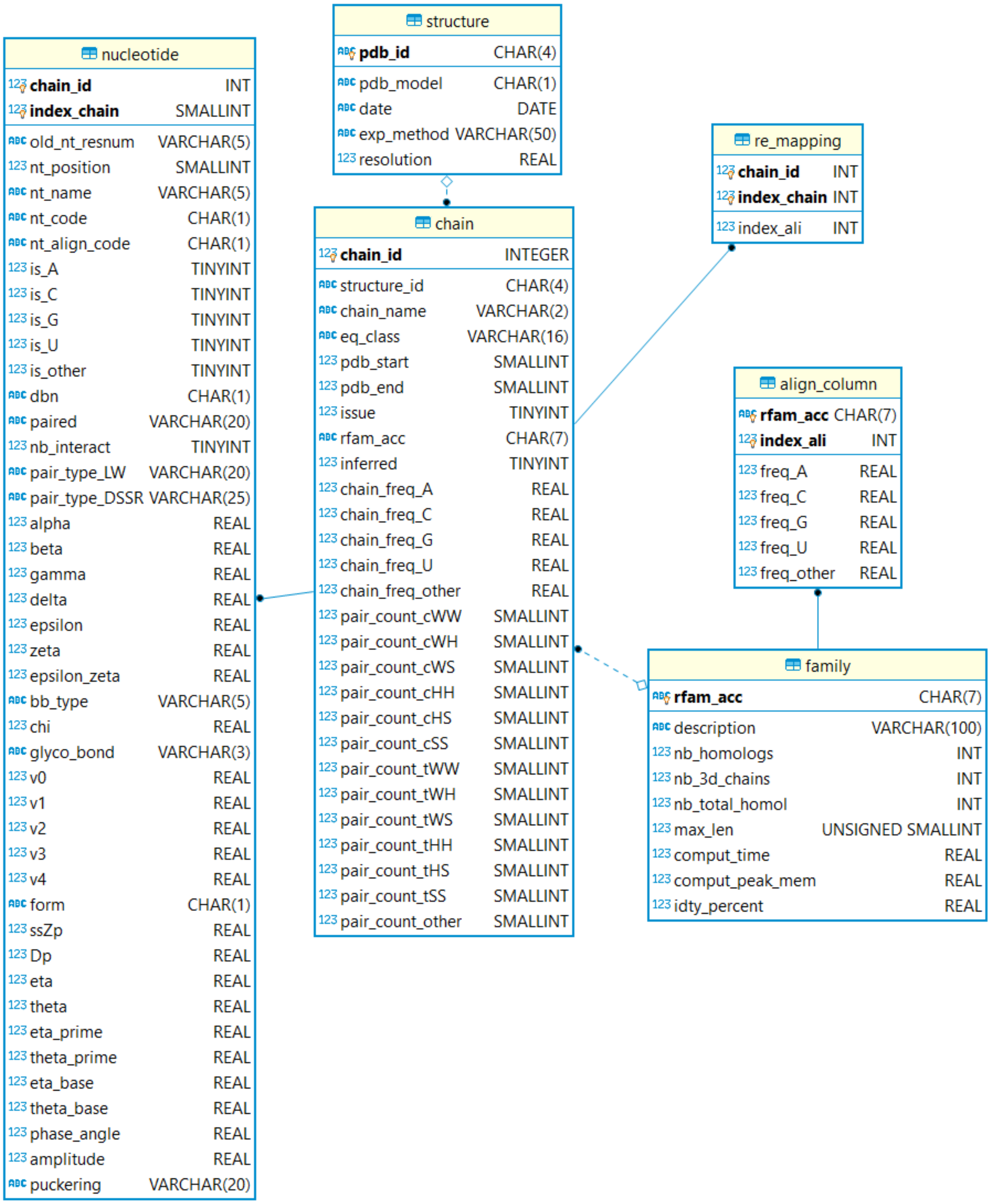

Figure 1: Database schema with table names and their respective data fields.

2 Rfam families used

Below in Table 2 are the content of the "family" table of the database. You can reproduce this with the following SQL query:

```
SELECT rfam_acc, nb_homologs, nb_3d_chains, nb_total_homol, max_len, idty_percent, description
FROM family ORDER BY nb_3d_chains DESC;
```

Table 2: Number of RNA sequences mapped to Rfam families. This data results from the Rfam-PDB mappings, extended to RNA equivalence classes as given by the BGSU redundancy list. For ribosomal RNAs, the number of sequence hits reported is the number of sequences included in their ARB database files: 2 225 272 for SSUs (ARB file release 138) and 198 843 for LSUs (ARB file release 132).

Rfam accession	Nb seq. hits (Rfam & Silva)	Nb 3D chains (from PDB)	Total	Maximum seq. length	% identity	Description
RF00005	1429931	1694	1431625	294	0.56	tRNA
RF00001	140644	1121	141765	346	0.69	5S ribosomal RNA
RF00177	2225272	976	2226248	3532	0.71	Bacterial small subunit ribosomal RNA
RF02541	198843	880	199723	8886	0.79	Bacterial large subunit ribosomal RNA
RF01960	2225272	317	2225589	5326	0.76	Eukaryotic small subunit ribosomal RNA
RF02543	198843	315	199158	11047	0.59	Eukaryotic large subunit ribosomal RNA
RF00002	198843	295	199138	290	0.68	5 8S ribosomal RNA
RF01852	2359	98	2457	113	0.43	Selenocysteine transfer RNA
RF02540	198843	72	198915	9020	0.96	Archaeal large subunit ribosomal RNA
RF00167	2631	54	2685	157	0.65	Purine riboswitch
RF01846	643	46	689	538	0.27	Fungal small nucleolar RNA U3
RF00026	47589	45	47634	432	0.63	U6 spliceosomal RNA
RF00234	935	42	977	381	0.79	glmS glucosamine phosphate activated ribozyme
RF00020	7673	39	7712	189	0.59	U5 spliceosomal RNA
RF00004	16770	34	16804	343	0.57	U2 spliceosomal RNA
RF00162	6026	30	6056	376	0.9	SAM riboswitch box leader
RF02001	3545	30	3575	341	0.93	Group II catalytic intron D1 D4 3
RF00028	2611	28	2639	893	0.7	Group catalytic intron
RF01998	2271	26	2297	152	0.76	Group II catalytic intron D1 D4 1
RF00059	12558	24	12582	256	0.79	TPP riboswitch THI element
RF00382	79	22	101	68	0.74	DnaX ribosomal frameshifting element
RF00504	4591	19	4610	250	0.93	Glycine riboswitch
RF00169	5208	17	5225	122	0.69	Bacterial small signal recognition particle RNA
RF01051	4682	17	4699	271	0.79	Cyclic di GMP riboswitch
RF00023	6693	16	6709	785	0.72	transfer messenger RNA
RF00168	2239	16	2255	335	0.96	Lysine riboswitch
RF00379	3855	16	3871	325	0.77	ydaO yuaA leader
RF01510	15	16	31	64	0.87	M florum riboswitch
RF00017	42477	15	42492	807	0.35	Metazoan signal recognition particle RNA
RF01763	647	13	660	83	0.98	Guanidine III riboswitch
RF00008	3180	12	3192	133	0.71	Hammerhead ribozyme type III
RF00015	7692	12	7704	311	0.65	U4 spliceosomal RNA
RF00032	30493	12	30505	89	0.77	Histone UTR stem loop
RF01344	379	12	391	36	0.99	CRISPR RNA direct repeat element
RF01831	637	12	649	250	0.82	THF riboswitch
RF01959	2225272	12	2225284	3682	0.23	Archaeal small subunit ribosomal RNA
RF00458	16	11	27	216	0.63	Cripavirus internal ribosome entry site IRES
RF00044	5	10	15	244	0.96	Bacteriophage pRNA

Rfam accession	Nb seq. hits (Rfam or Silva)	Nb 3D chains (PDB)	Total	Maximum seq. length	% identity	Description
RF00080	815	10	825	242	0.95	yybP ykoY manganese riboswitch
RF02679	72	10	82	80	0.98	Pistol ribozyme
RF00050	4079	8	4087	348	0.98	FMN riboswitch RFN element
RF01750	1625	8	1633	204	0.75	ZMP ZTP riboswitch
RF00029	15721	7	15728	342	0.59	Group II catalytic intron
RF00061	80	7	87	262	0.77	Hepatitis virus internal ribosome entry site
RF02545	2225272	7	2225279	629	0.23	Trypanosomatid mitochondrial SSU rRNA
RF00010	6481	6	6487	813	0.85	Bacterial RNase class A
RF00100	15277	6	15283	637	0.47	7SK RNA
RF00488	40	6	46	825	0.95	Yeast U1 spliceosomal RNA
RF02796	13	6	19	71	1.0	Pab160 RNA
RF00375	147	5	152	120	0.4	HIV primer binding site PBS
RF01734	2017	5	2022	160	1.0	Fluoride riboswitch
RF02012	1048	5	1053	192	0.57	Group II catalytic intron D1 D4 7
RF00009	1353	4	1357	1030	0.52	Nuclear RNase P
RF00011	790	4	794	437	0.42	Bacterial RNase class B
RF00037	1877	4	1881	57	1.0	Iron response element I
RF00373	373	4	377	635	1.0	Archaeal RNase P
RF00634	1244	4	1248	171	1.0	S adenosyl methionine SAM riboswitch
RF01689	211	4	215	216	0.9	AdoCbl variant RNA
RF01739	1102	4	1106	274	1.0	Glutamine riboswitch
RF01854	1143	4	1147	303	0.94	Bacterial large signal recognition particle RNA
RF00233	49	3	52	88	0.91	Tymovirus Pomovirus Furovirus tRNA like UTR
RF00254	522	3	525	90	0.8	mir 16 microRNA precursor family
RF00380	1058	3	1061	283	1.0	ykoK leader
RF02348	77	3	80	106	1.0	Trans activating crRNA
RF00027	1503	2	1505	97	0.8	let microRNA precursor
RF00174	14211	2	14213	477	0.58	Cobalamin riboswitch
RF00228	23	2	25	575	1.0	Hepatitis virus internal ribosome entry site IRES
RF00390	7	2	9	23	1.0	UPSK RNA
RF01704	670	2	672	95	1.0	Downstream peptide RNA
RF01725	792	2	794	159	0.96	SAM IV variant riboswitch
RF01786	629	2	631	106	1.0	Cyclic di GMP II riboswitch
RF02546	198843	2	198845	573	0.92	Trypanosomatid mitochondrial LSU rRNA
RF00003	15536	1	15537	306		U1 spliceosomal RNA
RF00013	3655	1	3656	255		6S SsrS RNA
RF00025	25	1	26	212		Ciliate telomerase RNA
RF00066	2972	1	2973	104		U7 small nuclear RNA
RF00102	130	1	131	175		VA RNA
RF00164	104	1	105	43		Coronavirus stem loop II like motif s2m
RF00209	26	1	27	281		Pestivirus internal ribosome entry site IRES
RF00250	72	1	73	61		Trans activation response element TAR
RF00442	856	1	857	227		Guanidine riboswitch
RF00505	21	1	22	66		RydC RNA
RF01807	12	1	13	219		GIR1 branching ribozyme
RF01826	18	1	19	94		SAM riboswitch
RF01857	344	1	345	344		Archaeal signal recognition particle RNA
RF02253	1199	1	1200	64		Iron response element II
RF02359	6	1	7	36		Bacteriophage MS2 operator hairpin
RF02519	6	1	7	34		ToxI antitoxin
RF02542	2225272	1	2225273	3056		Microsporidia small subunit ribosomal RNA
RF02553	192	1	193	189		Y RNA like
RF02680	34	1	35	104		PreQ1 III riboswitch
RF02683	234	1	235	188		NiCo riboswitch

3 Additional results

Figure 2 presents the number of Rfam families used considering structures of increasing resolutions for both found experimental methods.

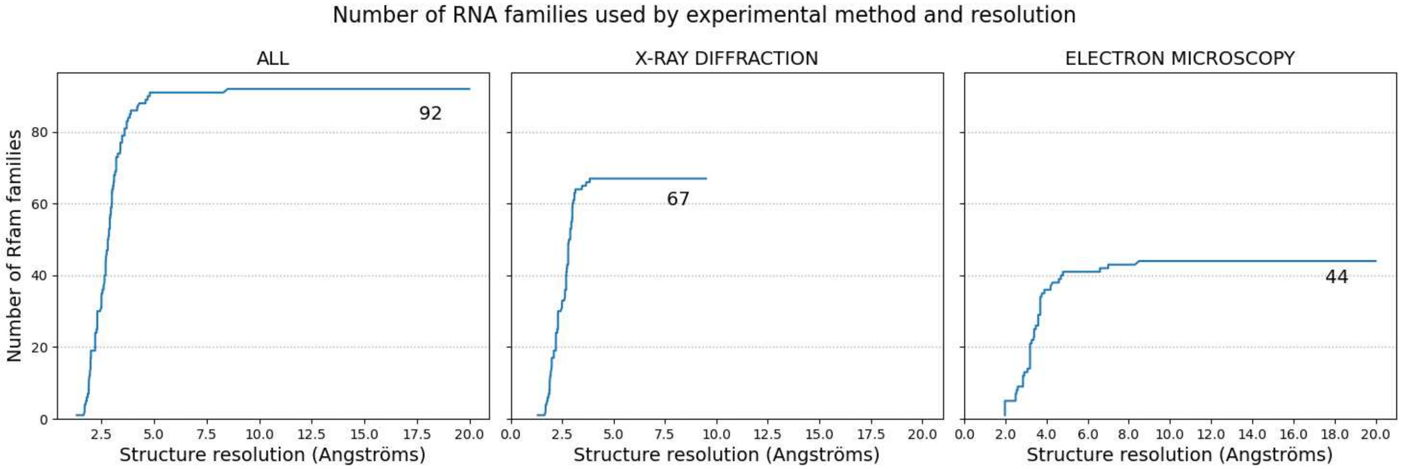

Figure 2: Number of Rfam families that have at least a representative 3D structure in our dataset when we accept structures of larger and larger resolutions. For X-ray diffraction, the curve stops early because there is no X-ray structure with resolution higher than 10.0 Angströms.

Figure 3 shows the number of solved chains in our dataset as a function of structure resolution and experimental method.

- **First column**: All the chains referenced in the BGSU non-redundant list are considered, even if they are not mapped to a Rfam family. As the BGSU list does not contain NMR structures, the RNANet database does not either. The distribution and the cumulative distribution are represented.
- **Second column**: Here we only consider chains with at least one mapping to a Rfam family. The distribution and the cumulative distribution are represented, plus a third curve, showing the fraction of the cumulative distribution composed by chains that are mapped only by inference using the BGSU lists. For example, chain 4BTC-A is not present in the Rfam-PDB mappings provided by Rfam. But, as it is part of the BGSU equivalence class `NR_4.0_35542.70`, whose members are known to be part of family RF00177 (the bacterial SSU rRNA), we can infer that 4BTC-A can be mapped to RF00177. The third curve shows the data gain in RNANet compared to the use of Rfam mappings only.
- **Third column**: Here we consider all the registered mapped chains, including multiple copies when a chain can be mapped to several families by inference. Inference is used only when no Rfam mapping is available. The distribution, cumulative distribution, and portion of the cumulative distribution obtained by inference only (and containing chain copies, if any) are represented.

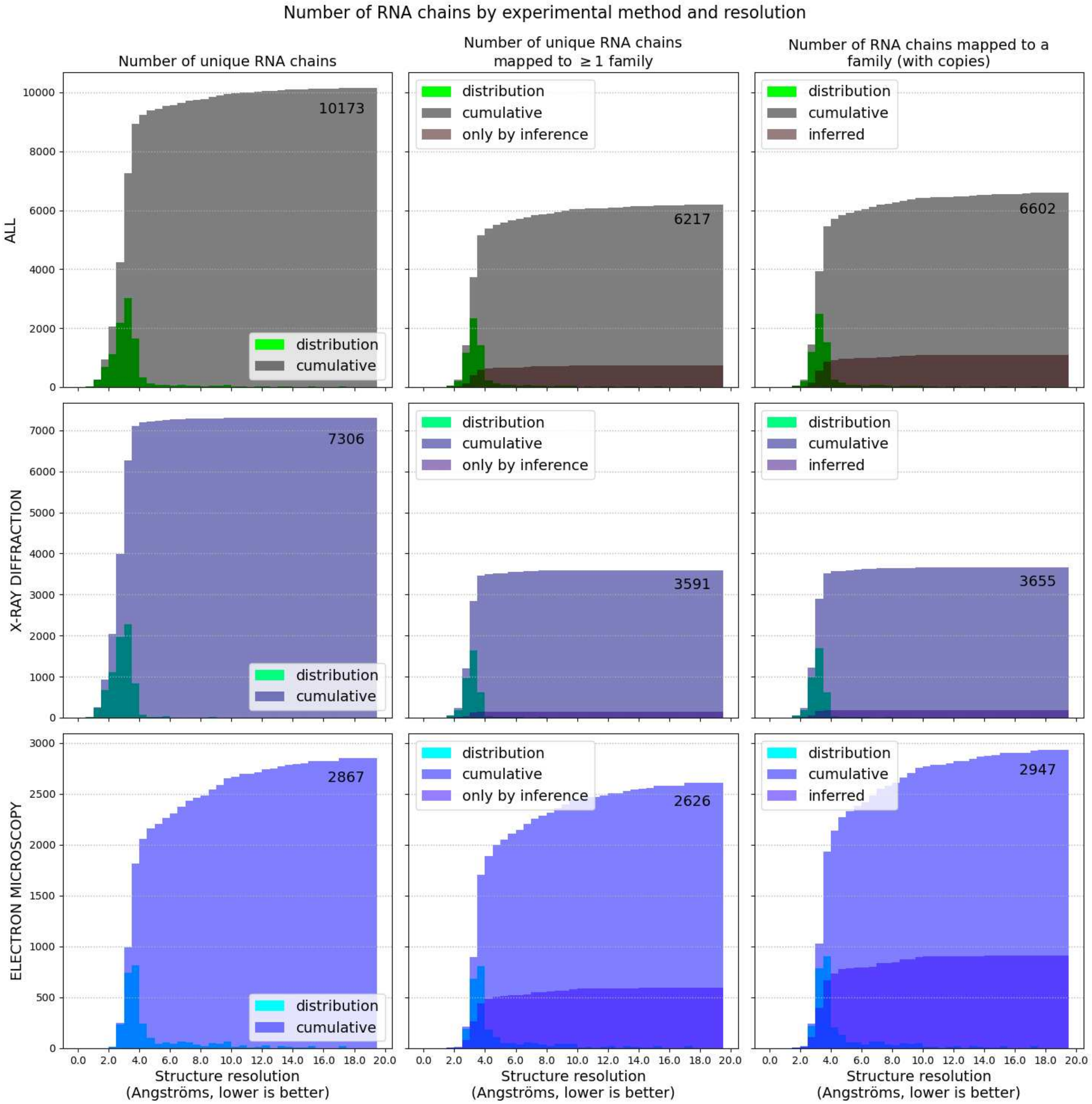

Figure 3: Distribution of the number of structures as a function of the initial PDB structure resolution. The top line summarizes all experimental methods merged, while the two following lines show the same plots, but only with chains solved by X-ray crystallography or electron microscopy, respectively.

Figure 4 presents statistics about base-base interactions, focusing on the Leontis-Westhof interaction type, and the two nucleotides sequence of the basepair. It shows that cis-Watson-Watson interactions are hugely more common that the other types, including of course the nucleotides from the stems. We can see that the majority of them are the so-called 'canonical' interactions cWW-AU, cWW-GU and cWW-GU (also called Wooble GU).

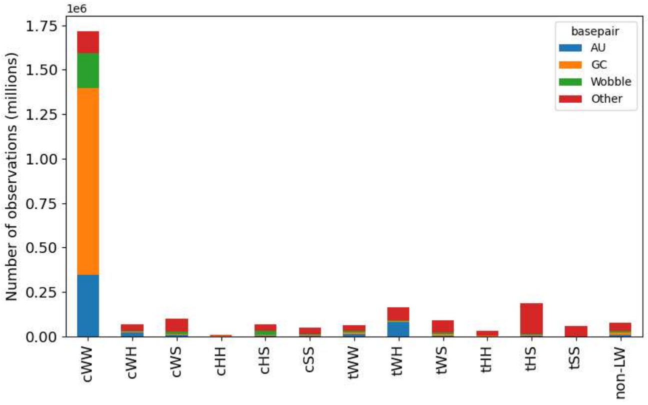

Figure 4: Count of the twelve Leontis-Westhof base pairing types in the dataset. Non-LW represents the base parings that do not fit the nomenclature. Interactions labelled cWW include the so-called canonical Watson-Crick ($G \equiv C$, $A = U$) and wobble base-pairs ($G = U$), represented in different colours.

4 Use case: reproduction of Wadley & al (2007) results

A data mining application of the RNANet dataset could be to reproduce, with the most recent data, the pseudotorsions joint distributions in a Ramachandran-like plot.

Wadley *et al.* proposed a first set of clusters observed in high-resolution structures available in 2007, separating nucleotides with riboses in C'2-endo or C'3-endo configurations. As a reminder, we extract their article's Figure 6 here in Figure 5. The left plot shows clusters concerning C3'-endo, non-helical nucleotides. The right plot shows clusters concerning C2'-endo nucleotides. Our Figure 6 reproduces their method with datasets of increasing resolution thresholds the scatter plots are superposed to Gaussian-kernel density estimates, the lowest level considered to delimit a cluster boundary being the average density of the plot plus one standard deviation. The more internal delimitations correspond to two and four standard deviations above the average density. The first lines A1 and A2 are expected to propose similar clusters compared to Figure 5 left and right plots, respectively. The first column corresponds to unique RNA chains with resolution 2.0Å or better (less than 2.0), similar to Wadley's set. The scatter plot looks similar to Wadleys' figure, but several clusters are now considered not significant using their own methodology, falling under the threshold of one standard deviation. Increasing the dataset size to unique RNA chains with resolutions up to 3.0 and 4.0 Å, the clusters appear again for 2'-endo nucleotides, but not for 3'-endo non-helical nucleotides. The two last lines B1 and B2 use the η'/θ' pseudobond system instead of η/θ.

The right column (using structures with resolution $\leq$ 4.0 Å) corresponds to the main paper's Figure 5.

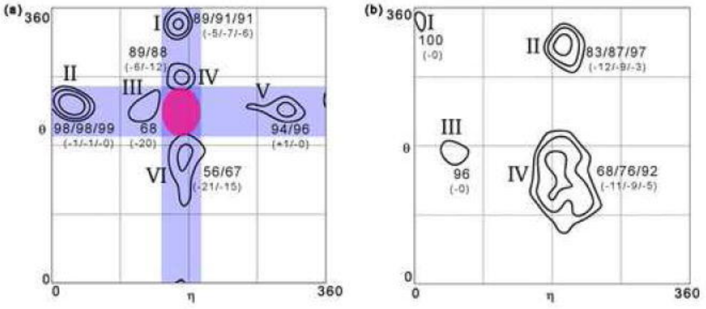

Figure 5: Figure 6 from Wadley LM, Keating KS, Duarte CM, Pyle AM. Evaluating and learning from RNA pseudotorsional space: quantitative validation of a reduced representation for RNA structure. *J Mol Biol.* 2007 Sep 28;372(4):942-957. Publicly available at Pubmed: https://pubmed.ncbi.nlm.nih.gov/17707400/

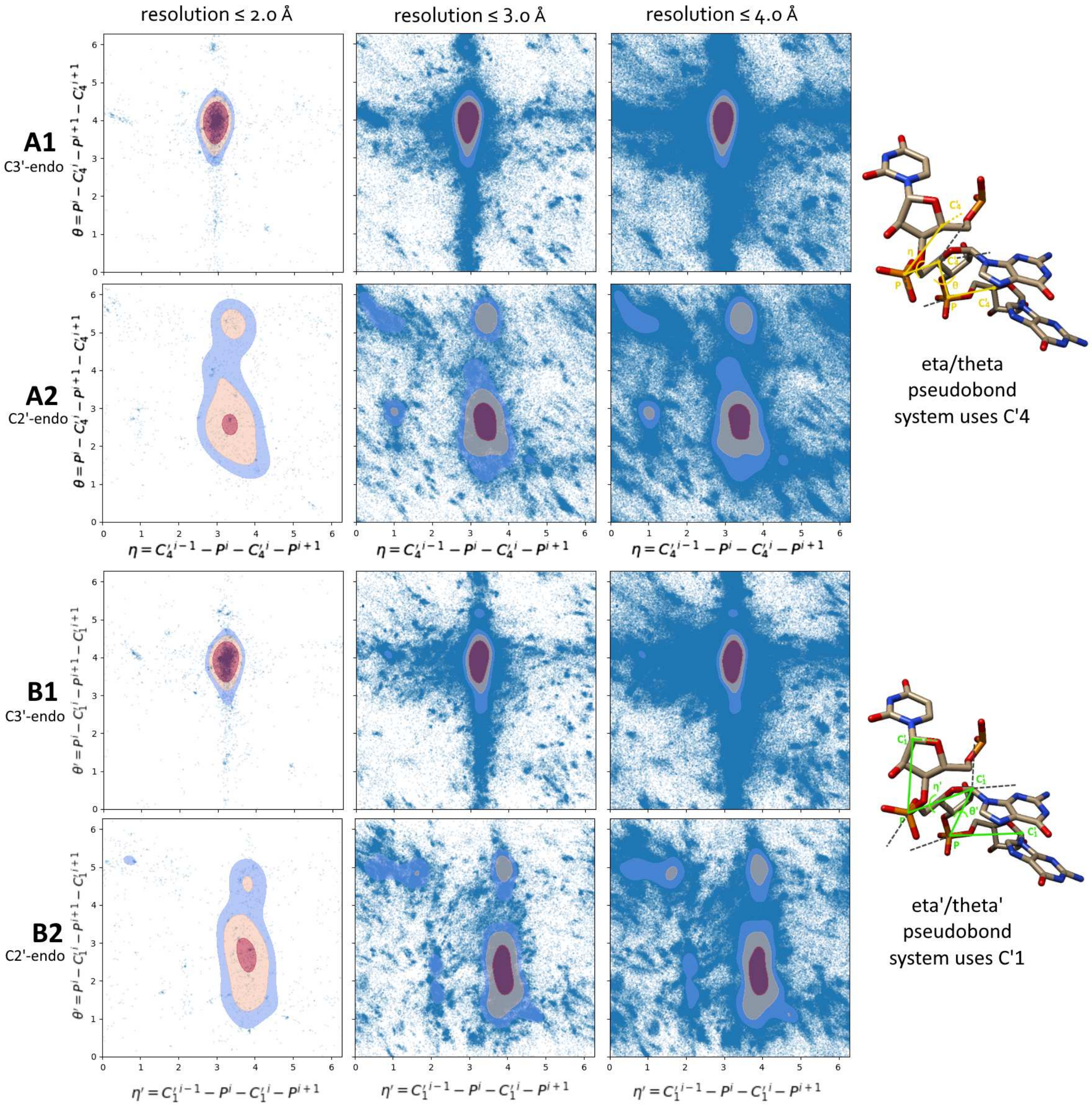

Figure 6: Ramachandran-like plots of the couples of pseudotorsions (η, θ) in A1 (non-helical C3'-endo nucleotides) and A2 (C2'-endo nucleotides), and (η', θ') in B1 (non-helical C3'-endo nucleotides) and B2 (C2'-endo nucleotides). Gaussian kernel density estimates are superposed to scatter plots. The line contours correspond to $\rho + \sigma$, $\rho + 2\sigma$ and $\rho + 4\sigma$ where ρ is the average height of the kernel and σ its standard deviation. Columns correspond to three different datasets of unique RNA chains of increasing resolutions. Including more structures comes at the cost of decreasing precision in the pseudotorsion values, because of the approximative atom positions in lower resolution structures.
